# Inhibitory activity of postbiotic produced by strains of *Lactobacillus plantarum* using reconstituted media supplemented with inulin

**DOI:** 10.1186/1757-4749-6-23

**Published:** 2014-06-14

**Authors:** Karwan Yassen Kareem, Foo Hooi Ling, Loh Teck Chwen, Ooi May Foong, Samsudin Anjas Asmara

**Affiliations:** 1 Department of Animal Science, Faculty of Agriculture, Universiti Putra Malaysia, 43400 UPM Serdang, Selangor, Malaysia; 2 Department of Animal Resource, University of Salah al- Din, Erbil, Iraq; 3 Department of Bioprocess Technology, Faculty of Biotechnology and Biomolecular Science, Universiti Putra Malaysia, 43400 UPM Serdang, Selangor, Malaysia; 4 Institute of Bioscience, Universiti Putra Malaysia, 43400 Serdang, Selangor, Malaysia; 5 Institute of Tropical Agriculture, Universiti Putra Malaysia, 43400 Serdang, Selangor, Malaysia

**Keywords:** *Lactobacillus plantarum*, Postbiotic, Inulin, Modified inhibitory activity

## Abstract

**Background:**

The present study aimed to determine the inhibitory activity of postbiotic produced by *L. plantarum* using reconstituted media supplemented with different levels of inulin and to select the best combination based on the modified inhibitory activity (MAU/mL) against pathogens.

**Methods:**

Postbiotics were produced by 6 strains of *L. plantarum* (RG11, RG14, RI11, UL4, TL1 and RS5) using reconstituted media supplemented with different levels of Inulin (0, 0.2, 0.4, 0.6, 0.8, and 1.0) yielding 36 combinations.

**Results:**

The combination of postbiotic and inulin had higher inhibitory activity than postbiotic alone against all indicator organisms except *Pediococcus acidilactici,* and *E. coli*. The RI11 + 0.8% Inulin, RG14 + 0.8% Inulin and RG14 + 0% Inulin had significantly (p < 0.05) higher MAU/mL against *P. acidilactici* than other treatments. The RI11 + 0.8% Inulin and RG14 + 0.4% Inulin had a significantly (p < 0.05) higher MAU/mL against VRE. The MAU/mL against *L. monocytogenes* was greater in RI11 + 1.0% Inulin, RI11 + 0.6% Inulin and RI11 + 0.8% Inulin. The combinations of RS5 + 1.0% Inulin, RS5 + 0.8% Inulin and RS5 + 0.6% Inulin had greater MAU/mL against *S. enterica;* whereas in *E. coli*, the inhibitory activity had higher activity that can only be found in RS5 + 0.8% Inulin.

**Conclusion:**

Combination of postbiotics and inulin which had higher optical density tends to have lower pH which corresponds to increased inhibitory activity against indicator organisms. The results of this study show that postbiotics and inulin supplementation enable to inhibit proliferation of pathogenic bacteria.

## Background

The act of feeding antibiotics to livestock has been practiced for over fifty years [1]. The mode of action of antibiotics is that they alter microbial metabolism thereby suppressing the growth of pathogenic microbes in the gut [2]. However, the use of antibiotics has been criticised for having negative impacts on animal production and health as it could have residual effects on tissues long after withdrawal. Furthermore, microbial resistance [3], genotoxicity and allergies [4] are other problems caused by the use of antibiotics in the animals.

Moreover, bacteria cause such problems as food poisoning and diarrhea. The bacteria considered as the main cause for food poisoning are *L. monocytogenes, Campylobacter*, *Salmonella*, and pathogenic *E. coli*. One of the most popular disease caused by food-borne bacteria worldwide is *Salmonella,* which is an important pathogen found in food produced by animals. This type of pathogen usually becomes widespread by trade in non-heated food products made from animal meat. The microbial strains which show resistance to antimicrobials, usually, as a result of antimicrobial procedure in animals, cause hazardous problems for public health [5].

Because of these consequences, there is increasing public awareness and pressure to search for alternatives to antibiotics [6,7]. Prebiotics, probiotics, postbiotics, and medicinal plants are common natural feed additives recently used in poultry industries to promote the immune response and the performance of birds. Postbiotics are substances produced in the final or intermediate stage of metabolic process in Lactic acid bacteria, while prebiotics are defined as indigestible carbohydrates that leave a desired effect on the host by selective growth stimulation or activation of one or more beneficial bacteria in a large part of the gastrointestinal tract [8]. Recently, various findings have reported that postbiotic possesses myriad beneficial probiotic effects on the growth of animals and particularly the gut health when used as additive in animal diet [9-11]. One of the features of postbiotics is their ability to reduce pH value thereby inhibiting opportunistic pathogens in the feed and gut of animals. In addition, postbiotics display wide inhibitory activity against various species of pathogens such as *Listeria monocytogenes, Clostridium perfringens, Salmonella enterica,* and *Escherichia coli*[12-15].

Various studies have been conducted to test the individual efficacy of postbiotics and prebiotics separately. However, no study has been conducted using the combination of prebiotics and postbiotics. Since most postbiotics exhibit probiotic effect, there could be a synergy between a prebiotic and a postbiotic. Thus, the present study was conducted to determine the inhibitory activity of postbiotic produced by 6 strains of *L. plantarum* using reconstituted media supplemented with different levels of inulin (a prebiotic) and to select the best combination based on the modified inhibitory activity against pathogens and an indicator bacterium.

## Methods

### Reviving culture

#### Postbiotic producer

RG11, RG14, RI11, UL4, TL1, and RS5 as *Lactobacillus plantarum* used in this study were previously isolated from Malaysian fermented food [16,17] and kept at -20°C in MRS broth containing 20% (v/v) glycerol. The stock cultures were revived twice in de-Mann Rogosa Sharpe (MRS) broth and incubated at 30°C for 48 and 24 hrs subsequently at static condition. Plate spreading was then conducted for the revived cultures, followed by 48 hrs of incubation. A single colony was picked and inoculated into 10 mL MRS broth and incubated for 24 hrs, followed by re-sub-culturing into 10 mL MRS broth and again incubating for 24 hrs. The culture was then ready to be used as an inoculum for the fermentation.

### Indicator microorganism

In this study, *Pediococcus acidilactici* 4–46 was chosen as the indicator due to the fact that it is a common food spoilage bacterium in food products for both humans and animals [18]. The preparation of culture was same as listed in the preparation of the postbiotic producer.

### Pathogenic bacteria

The reviving steps of *Listeria monocytogenes* L-MS, *Salmonella enterica* S-1000, *Escherichia coli* E-30 and Vancomysin Resistant *Enterococci* (VRE) are same as the postbiotic producer, except that nutrient media was used for the cultivation of VRE and *S. enterica*, incubated at 37°C and 30°C, respectively. *E. coli* was cultivated in LB broth at 37°C while *L. monocyotgenes* was cultivated at 30°C in Listeria Enrichment media. All the cultivation was performed under the agitation speed of 150 rpm.

### Media preparation

In this study, the reconstituted media of *L. plantarum* RG11, RG14, RI11, UL4, TL1 and RS5 were prepared for the production of postibiotic according to their composition. They were also mixed with different levels of inulin (0.2%, 0.4%, 0.6%, 0.8% and 1.0%), (w/v) before autoclaved at 118°C for 15 min.

### Production of postbiotic by *L. plantarum* strains

1% (v/v) of inoculum was inoculated into the respective reconstituted media supplemented with different levels of inulin, and incubated at static condition at 30°C. The postbiotic was collected after separating the bacterial cell by centrifugation at 10,000 × g for 15 min and used for analysis.

### Analysis

#### Agar well diffusion assay

The inhibitory activity of the produced postbiotics were tested against indicator microorganism, *P. acidilactici* and pathogenic microorganisms; *L. monocytogenes, S. enterica*, VRE and *E. coli* using the Agar Well diffusion method [19]. A two-fold-serial dilution of postbiotic from 2^0^ to 2^5^ was conducted using 0.85% (w/v) NaCl solution. Each diluted postbiotic was inoculated at 20 μL into the corresponding well on pre-punched MRS agar plate for *P. acidilactici* and 100 μL into the pre-punched nutrient agar plate for *L. monocytogenes*, *S. enterica* and LB agar for *E. coli* while 60 μL inoculated into corresponding well on nutrient agar plate for VRE. The diameter of each well was 5.5 mm. The postbiotics were allowed to diffuse completely for 1 hr at room temperature before overlaid with 3 mL of corresponding soft agar inoculated with 1% (v/v) of *P. acidilactici*, *L. monocytogenes, S. enterica*, VRE, and *E. coli*, respectively. After incubation at 30°C for 24 hrs, the highest dilution factor with the clear zone’s diameter size larger than 0.1 cm of the initial diameter size was recorded. The diameter of the clear zone (mm) was measured and the modified bacteriocin activity was calculated based on the formula as shown below:

$$\mathrm{Modified}\;\mathrm{bacteriocin}\;\mathrm{activity}:\frac{\mathrm{The}\;\mathrm{highest}\;\mathrm{dilution}\;\mathrm{factor}}{\mathrm{Volume}\;\mathrm{of}\;\mathrm{postbiotic}\;\left(\mathrm{mL}\right)*\mathrm{diameter}\;\mathrm{of}\;\mathrm{zone}\;\left(\mathrm{mm}\right)}$$

### Optical density and pH determination

Optical density measured the turbidity of a suspension which reflects cell mass or number of a bacterial culture. 1 mL of culture from each treatment group was centrifuged at 10,000 × g for 15 min. The cell pellet was washed once with 0.85% (w/v) and the optical density was determined at 600 nm using spectrophotometer (Novaspec III, Biochrom, Cambridge, UK). The pH of postbiotics was determined using pH meter (Mettle-Toledo., England).

### Statistical analysis

The factorial ANOVA was used for data analysis in this study. Data obtained for the modified bacteriocin activity (MAU/mL), inhibitory zone, pH, and optical density were subjected to generalized linear model of SAS. Duncan multiple range test was used to compare the significant difference of means.

## Results and discussion

The modified inhibitory activity against indicator and pathogenic organisms of all the 36 combinations of postbiotics and inulin are presented in Table 1. There were differences of inhibitory activity of different postbiotics produced by reconstituted media supplemented with inulin against different indicator organisms. The treatments P3.I5 (RI11 + 0.8% Inulin), P2.I5 (RG14 + 0.8% Inulin), and P2.I1 (RG14 + 0% Inulin) had a significantly (p < 0.05) higher MAU/mL against *P. acidilactici* than other treatments. Treatments P3.I5 (RI11 + 0.8% Inulin), P2.I3 (RG14 + 0.4% Inulin), and P2.I5 (RG14 + 0.8% Inulin) had a significantly (p < 0.05) higher MAU/mL against VRE. The MAU/mL against *L. monocytogenes* were greater in P3.I6 (RI11 + 1.0% Inulin), P3.I4 (RI11 + 0.6% Inulin), and P3.I5 (RI11 + 0.8% Inulin). The P6.I6 (RS5 + 1.0% Inulin), P6.I5 (RS5 + 0.8% Inulin), and P6.I4 (RS5 + 0.6% Inulin) had greater MAU/mL against *S. enterica.* For the *E. coli*, inhibitory activity was detected within only RS5, where the treatment P6.I5 (RS5 + 0.8% Inulin), P6.I1 (RS5 + 0% Inulin), and P6.I6 (RS5 + 1.0% Inulin) had higher MAU/mL activity.

**Table 1:** Modified bacteriocin activity (MAU/ml) score rank of 36 combinations of postbiotics produced by using reconstituted media supplemented with different levels of inulin against pathogens

**Treatments**	***P. acidilactici***		**VRE**		***L. monocytogenes***		***S. enterica***		***E. coli***		**Score**^**4**^
	**MAU/mL**	**Rank**^**3**^	**MAU/mL**	**Rank**	**MAU/mL**	**Rank**	**MAU/mL**	**Rank**	**MAU/mL**	**Rank**	
P3^1^.I5^2^	7866.67 ± 133.33^a^	1	6488.84 ± 88.88^a^	1	2240.00 ± 0.00^bc^	3	433.33 ± 3.33^g^	7	_	6	162
P3.I6	7200.00 ± 0.00^bc^	4	6044.40 ± 88.88^cd^	5	2453.33 ± 53.33^a^	1	433.33 ± 3.33^g^	7	_	6	157
P2.I5	7866.67 ± 133.33^a^	1	6399.96 ± 0.00^ab^	2	1226.66 ± 26.66^d^	5	193.33 ± 1.66^k^	12	_	6	154
P2.I1	7866.67 ± 133.33^a^	1	6399.96 ± 0.00^ab^	2	1226.66 ± 26.66^d^	5	186.66 ± 1.66^k^	13	_	6	153
P3.I1	7066.67 ± 133.33^c^	5	6222.18 ± 88.88^bc^	4	2186.66 ± 53.33^c^	4	380.00 ± 0.00^hi^	9	_	6	152
P3.I4	7200.00 ± 0.00^bc^	4	5688.85 ± 88.88^f^	9	2293.33 ± 53.33^b^	2	386.66 ± 3.33^f^	8	_	6	151
P3.I2	6800.00 ± 0.00^cde^	7	6222.18 ± 88.88^bc^	4	2186.66 ± 53.33^c^	4	380.00 ± 0.00^hi^	9	_	6	150
P2.I6	7466.67 ± 133.33^b^	2	6399.96 ± 0.00^ab^	2	1120.00 ± 0.00^de^	9	193.33 ± 1.66^k^	12	_	6	149
P2.I3	7333.33 ± 133.33^b^	3	6488.84 ± 88.88^a^	1	1146.66 ± 26.6^de^	8	170.00 ± 0.00^l^	14	_	6	148
P4.I5	7066.67 ± 133.33^c^	5	5066.63 ± 0.00^d^	10	1226.66 ± 26.66^g^	5	446.66 ± 3.33^f^	6	_	6	148
P6.I5	6266.67 ± 133.33^gh^	11	4888.86 ± 88.88^gh^	12	1200.00 ± 0.00^de^	6	813.33 ± 6.66^b^	2	153.33 ± 3.33^a^	1	148
P6.I6	6400.00 ± 0.00^fg^	10	4888.86 ± 88.88^gh^	12	1200.00 ± 0.00^de^	6	906.66 ± 6.66^a^	1	146.66 ± 3.33^abc^	3	148
P2.I4	7466.67 ± 133.33^b^	2	6222.18 ± 88.88^bc^	4	1173.33 ± 26.6^de^	7	170.00 ± 0.00^l^	14	_	6	147
P3.I3	6666.67 ± 133.3^def^	8	6044.40 ± 88.88^cd^	5	2186.66 ± 53.33^c^	4	373.33 ± 3.33^i^	10	_	6	147
P2.I2	7200.00 ± 0.00^bc^	4	6399.96 ± 0.00^ab^	2	1120.00 ± 0.00^e^	9	170.00 ± 0.00^l^	14	_	6	145
P6.I4	6266.67 ± 133.33^gh^	4	5066.64 ± 0.00^hi^	10	1200.00 ± 0.00^de^	6	786.66 ± 6.66^c^	3	136.66 ± 3.33^c^	5	145
P4.I6	6666.67 ± 133.3^def^	8	4977.75 ± 88.88^gh^	11	1200.00 ± 0.00^de^	6	446.66 ± 3.33^f^	6	_	6	143
P6.I2	6400.00 ± 0.00^fg^	10	4799.97 ± 0.00^hi^	13	1200.00 ± 0.00^de^	6	733.33 ± 6.6^d^	4	140 ± 0.00^bc^	4	143
P6.I1	6400.00 ± 0.00^fgh^	10	4622.19 ± 88.88^de^	15	1200.00 ± 0.00^de^	6	746.66 ± 6.66^e^	5	150 ± 0.00^ab^	2	142
P4.I1	6933.33 ± 133.33^cd^	6	4977.75 ± 88.88^gh^	11	1200.00 ± 0.00^de^	6	373.33 ± 3.33^i^	10	_	6	141
P4.I2	6933.33 ± 133.33^cd^	6	4888.85 ± 88.88^gh^	12	1200.00 ± 0.00^de^	6	373.33 ± 3.33^i^	10	_	6	140
P6.I3	6133.33 ± 133.33^gh^	12	4711.08 ± 88.88^i^	14	1200.00 ± 0.00^de^	6	786.66 ± 6.66^c^	3	136.66 ± 3.33^c^	5	140
P1.I1	6666.67 ± 133.3^def^	8	6399.96 ± 0.0^ab^	2	693.33 ± 13.33^f^	10	120.00 ± 0.00^m^	15	_	6	139
P4.I4	6666.67 ± 133.3^def^	8	4799.97 ± 0.00^c^	13	1200.00 ± 0.00^de^	6	380.00 ± 0.00^hi^	9	_	6	138
P1.I2	6666.67 ± 266.6^def^	8	6399.96 ± 0.00^ab^	2	693.33 ± 13.33^f^	10	110.00 ± 0.00^mno^	17	_	6	137
P1.I6	6400.00 ± 0.00^fg^	10	6399.96 ± 0.00^ab^	2	693.33 ± 13.33^f^	10	108.00 ± 1.66^mno^	18	_	6	134
P4.I3	6533.33 ± 133.3^efg^	9	4977.75 ± 88.88^gh^	11	1120.00 ± 0.00^e^	9	360.00 ± 0.00^j^	11	_	6	134
P1.I5	6533.33 ± 133.3^efg^	9	6222.18 ± 88.88^bc^	4	693.33 ± 13.33^f^	10	105.00 ± 0.00^no^	19	_	6	132
P5.I1	6666.67 ± 133.3^def^	8	6222.18 ± 88.88^bc^	4	586.66 ± 13.33^gh^	14	110.00 ± 0.00^mno^	17	_	6	131
P1.I3	6000.00 ± 0.00^h^	13	6399.96 ± 0.00^ab^	2	666.66 ± 13.33^fg^	11	108.00 ± 1.66^mno^	18	_	6	130
P5.I3	6000.00 ± 0.00^h^	13	6311.07 ± 88.88^abc^	3	600.00 ± 0.00^gh^	13	120.00 ± 0.00^m^	15	_	6	130
P5.I4	6000.00 ± 0.00^h^	13	6311.07 ± 88.88^abc^	3	586.66 ± 13.33^gh^	14	116.66 ± 1.66^mn^	16	_	6	128
P5.I2	6666.67 ± 133.3^def^	8	6222.18 ± 88.88^bc^	4	586.66 ± 13.33^gh^	14	100.00 ± 0.00°	22	_	6	126
P1.I4	6266.67 ± 133.3^fgh^	11	5955.51 ± 88.88^de^	6	640.00 ± 0.00^fgh^	12	103.00 ± 1.66°	20	_	6	125
P5.I6	6000.00 ± 0.00^h^	13	5866.63 ± 0.00^def^	7	600.00 ± 0.00^gh^	13	101.66 ± 1.66°	21	_	6	120
P5.I5	6000.00 ± 0.00^h^	13	5777.74 ± 88.88^ef^	8	573.33 ± 13.33^h^	15	103.33 ± 1.66°	20	_	6	118

^a-o^Means (mean of modified bacteriocin activity ± SEM) in the same column with common superscripts are non-significantly different. ^1^P1-P6 = different postbiotics (RG11, RG14, RI11, UL4, TL1 and RS5), which were numbered 1, 2, 3, 4, 5, 6. ^2^I1-I6 = Inulin levels (0, 0.2, 0.4, 0.6, 0.8 and 1%). ^3^Rank of modified bacteriocin activity against single indicator strain, ^4^Score is the sum of single indicator score as a subtraction of 36 and rank number (score = 36-rank). The treatment with higher score has stronger inhibitory activity against 5 above-mentioned indicator strains. It was arranged in descending order in the column.

The postbiotics produced by the 6 strains of *L. plantarum* used in this study exhibited broad antimicrobial activity and had the capacity to inhibit both gram positive and gram negative pathogens. This observation corroborates the findings of Sifour *et al.*[20], who reported that bacteriocin produced by *L. plantarum* F12 isolated from olive oil had broad inhibitory spectrum against *L. monocytogenese*. Similarly, Liasi *et al.*[13] observed that the antimicrobial agent produced by *L. plantarum* inhibited the growth of a range of gram-positive and gram-negative microorganisms such as *L. monocytogenes, E. coli*, *Staphylococcus aureus* and *Salmonella enterica*. The inhibitory effect, exhibited by the postbiotics and inulin combinations which were observed by the formation of clear and distinct zones around the wells, may be due to the presence of several antimicrobial compounds such as bacteriocins or organic acids [21]. Bacteriocin can be defined as proteineous compounds produced by bacteria, which exhibit bacteriostatic or bactericidal properties [14,22]. Bacteriocin from *L. plantarum* is a natural antimicrobial compound capable of inhibiting the growth of pathogens at molecular and cellular levels [23]. The protective effects of bacteriocin as food biopreservative and gut health have been demonstrated [24].

Organic acids act as an acidifying agent, reducing the pH of surrounding and survivability of non-acid-tolerant pathogens. During the production of postbiotic by *L. plantarum* strains*,* acetic and lactic acids are produced to promote the growth of producer cells [14,16]. High concentrations of organic acids and low pH can prevent the proliferation of food-borne pathogens and spoilage organisms [25,26]. In addition, the enzymatic activity of pathogens could be impaired by organic acids thus forcing the bacterial cell to utilize the remaining energy to oust excess proton H leading to the death of the bacteria [27]. Similarly, based on the mode of action of inulin, a prebiotic has been established. Dunkley *et al.*[28] and Rehman *et al.*[29] reported that the indirect antimicrobial effect of prebiotics could be due to production of fermentation products such as bacteriocin and short chain fatty acids capable of reducing pathogens by pH reduction. The production of short chain fatty acids (SCFAs) and bacteriocin capable of reducing pH has been reported as an indirect mechanism by which prebiotics such as inulin exert their antimicrobial influence [28]. According to Remesy *et al.*[30], fermentation of inulin and FOS leads to a considerable production of organic acids. It is also able to increase acidification of gut contents. Furthermore, prebiotics act as fermentation elements for particular members of the microbiota enhancing their numbers as well as the postbiotic of fermentation [31].

**Figure 1:**
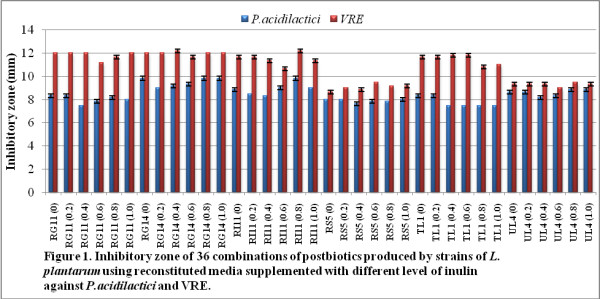
**Inhibitory zone of 36 combinations of postbiotics produced by strains of *L. plantarum *** **using reconstituted media supplemented with different levels of inulin against *P. acidilactici *** **and VRE.**

The inhibitory zone of postbiotic combinations against *P. acidilactici* and VRE is shown in Figure 1. The highest inhibitory zone against *P. acidilactici* was 9.83 mm in RG14 (0), RG14 (0.8), RG14 (1.0), and RI1 (0.8), whereas the highest inhibitory zone against VRE was 12.16 mm in RG14 (0.4) and RI11 (0.8).

**Figure 2:**
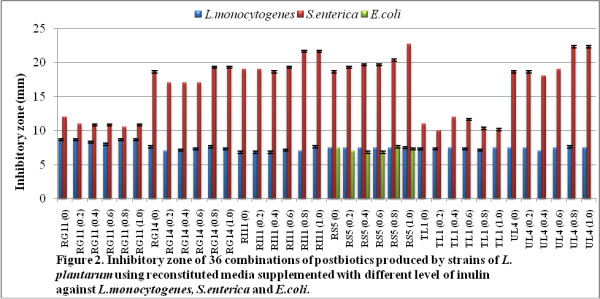
**Inhibitory zone of 36 combinations of postbiotics produced by strains of *L. plantarum *** **using reconstituted media supplemented with different levels of inulin against *L. monocytogenes, S. enterica *** **and *E. coli*.**

The inhibitory zone of postbiotic combinations against *L. monocytogenes, S. enterica,* and *E. coli* is shown in Figure 2. The highest inhibitory zone against *L. monocytogenes* was 8.66 mm in RG11 (0), RG11 (0.2), RG11 (0.8), and RG11 (1.0), whereas the highest inhibitory zone against *S. enterica* was 22.66 mm in RS5 (1.0). On the other hand, in *E. coli,* the inhibitory activity was detected just in RS5 in which the inhibitory zone of the combination RS5 (0.8) was 7.66 mm.

The optical density (OD_600_) and pH of various combinations of *L. plantarum* and inulin are shown in Table 2. There are significant differences (p < 0.05) in OD_600_ between different combinations of postbiotics and inulin. The mean optical density ranges from 1.92 to 2.28. The highest optical density observed in P6.I5 (RS5 + 0.8% Inulin). In contrast, the lowest OD was observed in P5.I6 (TL1 + 1.0% Inulin). As reported by Thu *et al*. [32], the differences in OD could be due to variation in the physiological and biochemical properties among different strains of *L. plantarum*. Choe *et al*. [1] also reported different strains of *L. plantarum* tend to grow and produce various levels of metabolite which may affect the value of the OD in similar condition. However, it was observed that combinations having higher OD tend to have lower pH. It was also observed that the combinations with low pH have high inhibitory activities against different indicator organisms. This observation was in line with the report of Fooks and Gibson [33] which suggests that low pH could be the probable mechanism of inhibitory action of the metabolites.

**Table 2:** **Optical density of different *L. plantarum *** **strains and pH of different postbiotic produced by using reconstituted media supplemented with different levels of inulin**

**Treatments**	**OD**	**pH**
P1^1^.I1^2^	2.06 ± 0.03^e^	4.05 ± 0.008^g^
P1.I2	2.02 ± 0.03^f^	4.12 ± 0.003^e^
P1.I3	1.99 ± 0.00^fg^	4.15 ± 0.008^d^
P1.I4	1.98 ± 0.003^g^	4.15 ± 0.003^d^
P1.I5	1.98 ± 0.003^g^	4.15 ± 0.003^d^
P1.I6	1.98 ± 0.003^de^	4.15 ± 0.005^g^
P2.I1	2.00 ± 0.00^f^	4.04 ± 0.003^e^
P2.I2	2.00 ± 0.003^fg^	4.06 ± 0.003^fg^
P2.I3	1.99 ± 0.003^fg^	4.06 ± 0.006^g^
P2.I4	1.99 ± 0.003^g^	4.07 ± 0.003^f^
P2.I5	2.0 ± 0.003^fg^	4.08 ± 0.00^f^
P2.I6	2.0 ± 0.003^de^	4.07 ± 0.003^g^
P3.I1	2.16 ± 0.006^d^	3.94 ± 0.01^h^
P3.I2	2.16 ± 0.003^d^	3.91 ± 0.006^i^
P3.I3	2.23 ± 0.005^bc^	3.91 ± 0.00^i^
P3.I4	2.23 ± 0.003^bc^	3.90 ± 0.003^i^
P3.I5	2.24 ± 0.003^ab^	3.87 ± 0.003^kl^
P3.I6	2.24 ± 0.00^ab^	3.87 ± 0.003^k^
P4.I1	2.20 ± 0.003^cd^	3.88 ± 0.003^k^
P4.I2	2.18 ± 0.006^d^	3.87 ± 0.005^k^
P4.I3	2.19 ± 0.006^cd^	3.84 ± 0.003^m^
P4.I4	2.20 ± 0.006^cd^	3.83 ± 0.00^m^
P4.I5	2.24 ± 0.003^b^	3.80 ± 0.0035^n^
P4.I6	2.20 ± 0.003^cd^	3.85 ± 0.00^l^
P5.I1	1.97 ± 0.003^gh^	4.34 ± 0.00^c^
P5.I2	1.94 ± 0.005^h^	4.37 ± 0.006^b^
P5.I3	1.94 ± 0.008^hi^	4.37 ± 0.003^ab^
P5.I4	1.94 ± 0.003^hi^	4.38 ± 0.010^ab^
P5.I5	1.93 ± 0.003^hi^	4.38 ± 0.01^a^
P5.I6	1.92 ± 0.003^i^	4.38 ± 0.005^ab^
P6.I1	2.25 ± 0.005^ab^	3.90 ± 0.003^ij^
P6.I2	2.26 ± 0.005^ab^	3.88 ± 0.005^jk^
P6.I3	2.26 ± 0.005^ab^	3.88 ± 0.003^k^
P6.I4	2.27 ± 0.005^ab^	3.87 ± 0.00^k^
P6.I5	2.28 ± 0.003^a^	3.85 ± 0.003^kl^
P6.I6	2.27 ± 0.003^ab^	3.85 ± 0.003^lm^

^a-n^Means (mean of OD and pH ± SEM) in the same column with common superscripts are non-significantly different. ^1^P1-P6 = different postbiotics (RG11, RG14, RI11, UL4, TL1 and RS5), which were numbered 1, 2, 3, 4, 5, 6. ^2^I1-I6 = Inulin levels (0, 0.2, 0.4, 0.6, 0.8 and 1%).

## Conclusion

It was evident in this study that postbiotic produced by *Lactobacillus plantarum* RG11, RG14, RI11, UL4, TL1, and RS5 using reconstituted media supplemented with different levels of inulin have the ability to inhibit various pathogens. Also, the combinations have a stronger inhibitory activity than the postbiotic alone due to the synergistic effect of postbiotic and inulin. The increase in optical density of the combinations contributed to a lower pH. Among the 36 treatments, P3.I5 (RI11 + 0.8% Inulin), P3.I6 (RI11 + 1.0% Inulin), and P2.I5 (RG14 + 0.8% Inulin) showed a higher level of modified bacteriocin activity. The results of this study show that postbiotics and inulin supplementation enable to inhibit proliferation of pathogenic bacteria.

## Competing interests

The authors declare that they have no competing interests.

## Authors’ contributions

FHL and LTC provided probiotic strains and method to produce postbiotic. KYK and MFO performed inhibitory tests. KYK, LTC, FHL, MFO and SAA contributed to the writing of the manuscript. All authors read and approved the final manuscript.
